# Reverse Electrochemical Sensing of FLT3-ITD Mutations in Acute Myeloid Leukemia Using Gold Sputtered ZnO-Nanorod Configured DNA Biosensors

**DOI:** 10.3390/bios12030170

**Published:** 2022-03-10

**Authors:** Ramesh Thevendran, Kai Loong Foo, Mohd Hazwan Hussin, Emmanuel Jairaj Moses, Marimuthu Citartan, Haarindraprasad Rajintra Prasad, Solayappan Maheswaran

**Affiliations:** 1 Infectomics Cluster, Advanced Medical & Dental Institute, Universiti Sains Malaysia, Bertam, Kepala Batas 13200, Malaysia; rthevendran@student.usm.my (R.T.); citartan@usm.my (M.C.); 2 Nano Biochip Research Group, Institute of Nano Electronic Engineering, Universiti Malaysia Perlis, Kangar 01000, Malaysia; klfoo@unimap.edu.my; 3 Materials Technology Research Group (MaTReC), School of Chemical Sciences, Universiti Sains Malaysia, Minden 11800, Malaysia; mhh@usm.my; 4 Regenerative Medicine Cluster, Advanced Medical and Dental Institute, Universiti Sains Malaysia, Bertam, Kepala Batas 13200, Malaysia; emmanuel_jm@usm.my; 5 Faculty of Engineering and Computer Technology, AIMST University, Semeling 08100, Malaysia; 6 Department of Biotechnology, Faculty of Applied Sciences, AIMST University, Bedong 08100, Malaysia; 7 Centre of Excellence for Nanobiotechnology & Nanomedicine (CoExNano), Faculty of Applied Sciences, AIMST University, Bedong 08100, Malaysia

**Keywords:** acute myeloid leukemia, DNA biosensor, FLT3-ITD, Au sputtered-ZnO nanorods, electrochemical impedance spectroscopy

## Abstract

Detection of genetic mutations leading to hematological malignancies is a key factor in the early diagnosis of acute myeloid leukemia (AML). FLT3-ITD mutations are an alarming gene defect found commonly in AML patients associated with high cases of leukemia and low survival rates. Available diagnostic assessments for FLT3-ITD are incapable of combining cost-effective detection platforms with high analytical performances. To circumvent this, we developed an efficient DNA biosensor for the recognition of AML caused by FLT3-ITD mutation utilizing electrochemical impedance characterization. The system was designed by adhering gold-sputtered zinc oxide (ZnO) nanorods onto interdigitated electrode (IDE) sensor chips. The sensing surface was biointerfaced with capture probes designed to hybridize with unmutated FLT3 sequences instead of the mutated FLT3-ITD gene, establishing a reverse manner of target detection. The developed biosensor demonstrated specific detection of mutated FLT3 genes, with high levels of sensitivity in response to analyte concentrations as low as 1 nM. The sensor also exhibited a stable functional life span of more than five weeks with good reproducibility and high discriminatory properties against FLT3 gene targets. Hence, the developed sensor is a promising tool for rapid and low-cost diagnostic applications relevant to the clinical prognosis of AML stemming from FLT3-ITD mutations.

## 1. Introduction

**Figure 1:**
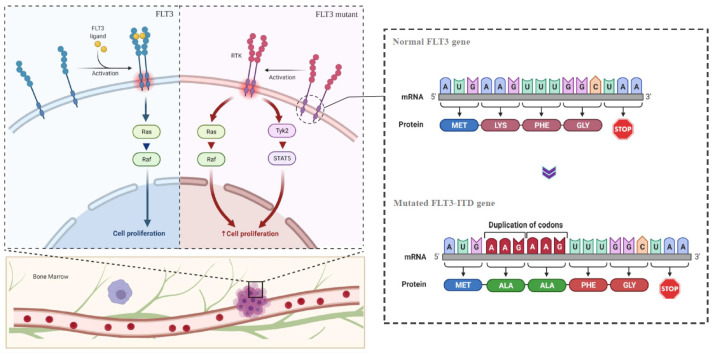
Diagram showing the difference between normal and mutated FLT3 genes with internal tandem duplications leading to ligand-independent activation and dimerization of receptor tyrosine kinase which causes uncontrolled cell proliferation and myeloid carcinoma.

Acute myeloid leukemia (AML) is a widely studied case of hematological malignancy involving the uncontrolled proliferation of myeloid progenitors with impeded differentiation capabilities [1]. Among the several different mutations associated with AML progressions, genetic alterations occurring in the FMS-like tyrosine kinase 3 (FLT3) gene is the most common known genetic marker diagnosed in both adult and pediatric AML patients [2,3]. Mutation in the FLT3 gene, encoding the membrane-bound receptor tyrosine kinase (RTK), disrupts such protein’s native function, leading to overexpressions, continuous clonal expansions, and damage towards the mechanisms of hematopoiesis relevant to cell differentiation and survival [4]. FLT3 mutations are categorized into two sub-types; internal tandem duplications (ITD) in the gene that encodes the Juxtamembrane (JM) domain and point mutations in the gene that encodes the tyrosine kinase domains (TKD). FLT3-ITD is observed to possess a higher frequency of incidence (approximately 25% of reported AML cases) and greater clinical significance compared with FLT3-TKD [5]. FLT3-ITD constitutes faithful, in-frame duplications of variable nucleotide lengths which deliver poor functionality to the JM domain, rendering the self-inhibitory system of the membrane protein disordered. The resulting changes negatively affect the activation of the protein, leading to auto-phosphorylation and, therefore, signal-independent cellular proliferation (Figure 1) [4,5]. Additionally, the co-occurrence of FLT3-ITD with other mutations such as the mutations of nucleophosmin (NPM1) and DNA methyltransferase 3A (DNMT3A) are further reported [6,7], indicative of their importance as excellent genetic biomarkers for early and relapse diagnosis of AML in patients.

Current approaches in identifying the FLT3-ITD mutation profile of AML patients are primarily restricted towards cytogenetic assessments—gene karyotyping [8], whole-genome sequencing [9,10], and PCR amplification assays [11,12]—to comprehend the different genotypic changes or mutations present in such cases [13,14]. The drawbacks of such techniques include longer turnaround times and complicated testing protocols. Aptasensors using aptamers to recognize AML-linked cell surface biomarkers have garnered substantial attention for their sensor-based usage, which provides rapid and cost-effective manners of AML diagnosis [15,16,17]. However, the application of DNA-based sensors capable of targeting and recognizing the genetic/gene mutations contributing to AML has not been expanded upon. Hence, in this work, we presented the development of an electrochemical DNA biosensor system designed for rapid and accurate diagnosis of AML caused by FLT3-ITD mutations. The developed biosensor will be a better substitute for existing conventional techniques as it combines the capability of methods such as sequencing or karyotyping to identifying genetic mutations with the usage of portable and rapid response properties of sensor-based systems.

The sensor adopts the use of interdigitated electrode (IDE) chips fabricated with an aptitude for sensitive recognition of the FLT3-ITD by employing a reverse-detection principle. IDE-based biosensors were chosen due to miniaturized nature of the device that offers multiple benefits such as portability, ease of modifications to the electrode surface, good reproducibility, low-cost, and reliable signal readouts [18]. Furthermore, the utilized IDE chip constitutes a nano-gapped electrode architecture which provides better sensitivity for nanomolecular analyte detections and faster signal responses compared with micro-gapped IDE chips or large cells of electrochemical measurements [19,20]. The system is crafted by coating the IDE chip’s active sensing region with gold (Au)-sputtered ZnO-nanorods. Here, ZnO is preferred due to its unique feature of possessing a wider band gap of 3.37 eV, which enables the transducer layer to sustain larger electric fields compared with other metal oxides, leading to higher breakdown voltage and better stability of the semiconductor [21,22]. The additional growth of the ZnO film into nanorod structures (ZnO nanorods) creates a larger surface area for bioreceptor functionalization, further improving the analytical sensitivity of the sensor platform [21,23,24,25]. Au sputtering of the sensing matrix also serves two key functions. In connection with increasing the structural integrity and conductivity of the ZnO nanorods [26], Au dopants additionally impart better chemisorptive properties to the chip’s surface, providing strong biointerfacing with thiol-modified DNA capture probes that operate as the bioreceptor element specifically targeting FLT3-ITD DNA mutations. The design and material composition of the modified sensor’s surface was analyzed to ensure a stable transducer layer efficient in deciphering minute changes in biochemical signals during analyte detection. The analytical parameters of the system such as their detection capacity, system linearity, sensitivity, stability, and reproducibility were further validated using electrochemical impedance spectroscopy (EIS).

## 2. Methods and Materials

### 2.1. In-Silico Analysis of FLT3 Gene Mutation for Probe Derivations

The FLT3 gene sequence was obtained from NCBI database (BC144039.1). The region of the gene encoding the juxtamemebrane domain of the tyrosine kinase 3 protein was isolated and selected to design a complementary sequence of 100 bp/nts in length as the desired AML-targeting DNA probe. Template sequence and primers modified with adapter sequences were further designed to build the complete probe in a step-wise manner through PCR amplifications (Table 1).

### 2.2. Preparation of theProbe Sequence

The DNA probe was synthesized using PCR reactions. PCR was performed using a mix of 25 μL of DreamTaq Green PCR master mix purchased from ThermoFisher Scientific (Waltham, MA, USA), 3 μL of 10 μM forward and reverse primers each, 15 μL of nuclease-free water, and 1 μg of template at 35 cycles using S1000 Thermocycler system (BioRad, Hercules, CA, USA). PCR cycles began at 95 °C for 3 min, then followed by 35 cycles of denaturation at 95 °C for 30 s, annealing at 55 °C for 30 s, and extension at 72 °C for 30 s. The resulting amplicons were analyzed using agarose gel electrophoresis methods with 4% agarose gel, 1× Tris-Acetate-EDTA (TAE) buffer, and Ethidium Bromide stains, run in a horizontal electrophoresis chamber system (BioRad, Hercules, CA, USA) at 100 V for 1 h. The amplified bands for each sample were observed and the UV image of the gel was captured and visualized. The double-stranded (ds) DNA amplicons were subjected to ethanol precipitation (2.5× absolute ethanol, 0.1× 3 M NaAc). The concentrated dsDNA templates were converted to ssDNA by denaturation using 8 M urea loading dye at 95 °C. Following UV imaging of the gel, the resulting band of the expected size was excised and purified using 12% PAGE. The concentration of the ssDNA probes was measured at 260 nm.

### 2.3. Extraction of Genomic DNA (gDNA) from Cultured Cancer Cell Lines

AML cancer cells were cultured under stringent conditions according to ATCC recommendations, at 37 °C in the presence of CO_2_. Once the cells reached a confluency of more than 80%, they were subjected to cell harvesting. The desired AML cell line and non-AML cell lines were collected for gDNA extractions. The collected cells were pelleted down at 1000 rpm for 10 min. The pellets were resuspended in 2 mL of 1× lysis buffer and vortexed well. These cells were then sonicated for 50 s repeatedly 3 times. The lysates were spun-down, and the supernatant was obtained. The gDNA was purified using Lucigen total DNA extraction kits. The obtained gDNA was further concentrated using ethanol precipitation and resuspended in DNase-free water. The gDNA was further used as the template sequence for asymmetric PCR (A-PCR) of FLT3 gene targets using 10 μL of 100 pmoles of reverse primers. The asymmetric PCR reaction mixture was used without any prior purification method.

### 2.4. Preparations of ZnO Sol-Gel Solution

Zinc acetate of 1.756 g was measured and added to 40 mL of molecular grade ethanol solution. The mixture was heated up to 60 °C and mixed at ~1000 rpm using a magnetic heat stirrer for 20 min. Monoethanolamine (MEA) stabilizers of 40.7 μL (1:1) were added at a time interval of 10 min (total 12 times) until the volume of monoethanolamine totaled up to 488 μL for 2 h. The solution was stirred constantly until a clear, transparent solution was obtained. The sol-gel was then left to cool/age in a dark area for at least 2–3 days.

### 2.5. Coating of ZnO Thin Films on IDE Chips

Interdigitated electrodes (IDE) chips were purchased (SilTerra Sdn. Bhd., Kulim, Malaysia). The chips were washed in ultrasonic baths cleaners and quickly rinsed with distilled water to remove dirt particles. The prepared ZnO Sol-Gel was spin-coated onto the chip’s sensing surface area using Laurell WS-650-Hzb-23B model spin coater. The initial spin speed was 700 rpm for 20 s followed by increasing the spin speed to 3000 rpm, 30 s, and stopping for 5 s to allow the layers to settle from the rotational movements. The wafers were then heated sequentially at 60 °C for 20 min, 150 °C for 10 min, and finally at 50 °C for 30 min to ensure complete evaporation of the remaining solvents. The coating process was repeated another two times to allow a total of three standard coating layers. The chips were then subjected to annealing at 300 °C for 2 h, allowing high crystallization of the ZnO film layers.

### 2.6. Hydrothermal Growth of ZnO-Nanorods with Gold Sputtering

ZnO-Nanorods were grown using hydrothermal growth methods described in Perumal et al. [27]. Zinc nitrate hexahydrate and hexamethylenetetramine were weighed to 1.487 g and 0.701 g respectively to obtain 0.025 M of both substances (1:1). Both chemicals were mixed in 400 mL of distilled water and stirred at ~1000 rpm at RT for 20 min. The ZnO film-coated chips are then dipped into the solution in an inverted position and incubated at 95 °C for 5 h to allow hydrothermal growth of uniform nanorods. The Nanorods were cleaned with isopropanol and deionized water to remove residual salts prior to annealing at 300 °C for 2 h for crystallizations. Gold sputtering was performed using Quorum 150R S for 40 s at 30 mA currents to obtain a thin film of Au layer on top the ZnO nanorods.

### 2.7. DNA Capture Probe Immobilization

The prepared ssDNA probe was immobilized on the Au-sputtered ZnO-Nanorods using duplexed-tagging strategies [28,29]. Equimolar amount of ssDNA probe and thiolated-poly(T) oligo strands were mixed and subjected to heating at 95 °C followed by rapid cooling for the duplex formation in the presence of 20 mM DTT. Ten µg of the thiolated ssDNA probes was added on top of the coated chip surface and left to incubate for 3 h or overnight within a wet chamber. The chips were then rinsed with LINA-T (0.05% Tween) to remove unbound probes and stored at 4 °C [28].

### 2.8. Surface Characterization of the Fabricated Sensor Surface

The designed sensing surface of IDE chips was subjected to field-emission scanning electron microscopy (FESEM), EDX spectrum, X-ray diffraction (XRD), and Fourier-transform infrared spectroscopy (FTIR) analysis to characterize and validate the material and morphological aspects of the derived sensor.

### 2.9. Target Hybridization and Gene Detection through EIS Measurements

EIS measurements were performed using Novocontrol alpha high-frequency analyzer (Hundsangen, Germany). Four individual EIS measurements were taken; surface-coated IDE, probe-immobilized IDE, and normal and mutated FLT3 gene target hybridized IDE. Each measurement was taken by immersing the chip’s active sensing area in 1× PBS buffer with 2 mM K_3_(Fe(CN)_6_)/K_4_(Fe(CN)_6_), dropped on top of the chips at RT. Intermediate washing steps were performed three times using PBST. The impedance spectra of the real and imaginary parts of impedance, Zs’ and Zs” respectively were obtained and recorded by sweeping the frequency of 0.1 Hz–100 kHz with an applied AC voltage of 10 mV RMS. Nonlinear curve fitting was performed for the obtained EIS results using Randles equivalent circuit using ZView. The obtained charge transfer resistance (R_ct_) values were subsequently recorded.

### 2.10. Evaluating the Analytical Performance of the AML Biosensor

Analytical performances such as the signal response, sensitivity, stability, and reproductivity were performed in a similar manner. The sensitivity of the fabricated AML biosensor was analyzed using a range of target DNA concentrations of 1 nM to 1 µM. The stability of the modified chips was also analyzed using the same coated IDE chips by measuring the EIS outputs and the respective R_ct_ values once a week for the next 4 weeks successively. Reproducibility of the detection signals was also analyzed by measuring the EIS signal outputs and R_ct_ values multiple times, at least 3–4 times during each analytical test using the same FLT3 gene target.

## 3. Results and Discussions

### 3.1. Detection Principles of the Designed Biosensor

**Figure 2:**
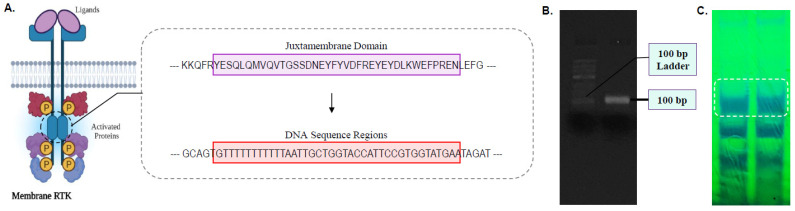
(**A**) The DNA region of the FLT3 gene which corresponds to the juxtamemebrane domain of membrane RTK where ITD occurs. (**B**) Gel-electrophoresis image showing the PCR bands of the FLT3 probe. (**C**) UV shadowing image of PAGE run using the PCR reaction mixture.

FLT3-ITD mutations exhibit various patterns of in-frame duplications, resulting in unpredictable nucleotide lengths ranging from three to more than 200 base pairs as well as more than one duplication fragment in AML patients [30,31]. Designing a single-stranded DNA-based capture probe capable of identifying FLT3-ITD mutations within clinical samples excludes practicality in AML diagnosis as such inconsistency in sequence duplications complicates hybridization of the probe sequence to targeted gene regions, therefore requiring the need to tediously design probes for each different mutation being studied. To circumvent this, we chose to design a single probe sequence capable of binding to the normal/unmutated form of the FLT3 gene. Since FLT3-ITD is reported to occur in the JM domain of membrane-bound RTK, both the amino acid sequence and the FLT3 genesequence were analyzed in tandem to isolate the sequence region of the gene which corresponds to the JM domain (Figure 2A). A complementary sequence was designed against the selected DNA region, serving as the desired ssDNA capture probe specific against the FLT3 gene. This manner of “reverse detection” whereby the capture probe targets an unmutated FLT3 gene sequence enables the proposed sensor system to effectively differentiate between normal and FLT3-ITD relevant AML samples based on the inability of the probe to hybridize with the mutated target sequence as compared to the normal target sequence. This creates a system that can be used to generally identify any form of genetic mutation associated with the JM region of the FLT3 gene, being independent of the different cell-line subtypes and ITD mutation frequencies found in such AML cases.

### 3.2. Derivation of FLT3-Targeting ssDNA Probe Sequence

Following the establishment of the detection logic underlying the sensor platform, the FLT3-targeting DNA probe was synthesized through a series of PCR amplification steps using primers modified with adapter sequences. The capture probe was also designed to include a poly(A)-tail at the 3′-end to enable duplex-tagging with thiolated poly(T)-tail sequences, which is required for surface functionalization of sensor chips with the desired capture probe. The size of the PCR amplicons was validated through gel electrophoresis to ensure a complete probe sequence was acquired. A band of 100 bp in size is visualized in Figure 2B, indicating the presence of a full-length probe incorporated with both the FLT3 complementary and poly(a) tail regions. In order to convert the double-stranded (ds) PCR amplicons, a heat-urea denaturation technique described by Hegedus et al. was utilized [32]. Subsequent to ethanol precipitation of the PCR DNA amplicons, the templates were heat denatured at 95 °C with 8 M urea, resulting in complete separation of the complementary helical strands, being incapable of re-association. The denatured templates were PAGE purified (Figure 2C) and extracted using the crush-and-soak method [33].

### 3.3. Confirming Amalgamations of Poly(A) Tail within the Capture Probe

**Figure 3:**
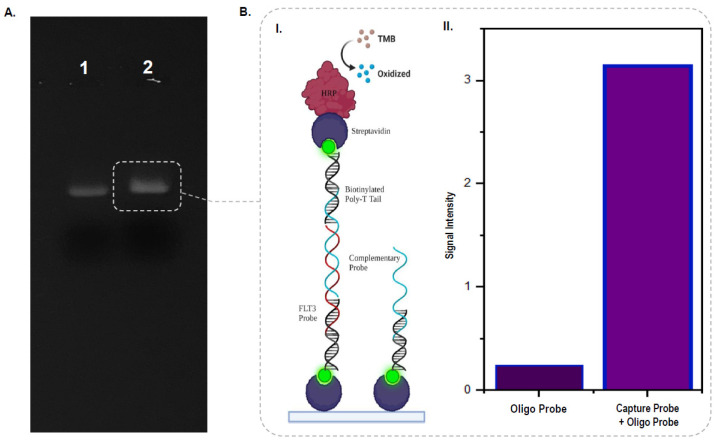
(**A**) Gel image showing the difference between ssDNA probe (lane 1) and poly(T) strand duplexed probe sequences (lane 2). (**B**(**I**)) Schematic representation of the ELISA assay used to confirm duplexed-tagging of the capture probe. (**B**(**II**)) Bar chart of the signal intensity measured in the presence and absence of the ssDNA capture probe.

Experimental validations revealed the DNA fragment extracted from the first band visualized in the PAGE run (Figure 2C) to be the FLT3 probe sequence of interest. Figure 3A exemplifies use of a gel-shift assay that portrays the changes in DNA bands observed in the presence of a complementary 21 bp biotinylated poly(T) sequence. Duplexing of the probe’s poly(A) tail section with the poly(T) oligo led to both increased band intensities due to an increased amount of EtBr intercalations at the helical regions and a slight shift in the position of the bands due to the additional molecular weight conferred by the biotinylated strands. Besides gel-shift assays, an ELISA-based assay was also conducted to further confirm the presence of the poly(A) tail section within the designed probe. Here, the assay applied the use of streptavidin-coated microtiter plates and a 51 bp oligo sequence complementary to the ssDNA probe’s target binding section. Incubation of the capture probe duplexed with biotin strands within the coated wells immobilized with the probe to the surface in proper orientations. Subsequent incubations with the complementary probe similarly duplexed with biotin strands yielded a helical assembly having free-end biotin molecules, as illustrated in the schematic representation of Figure 3(BI). With stringent intermediate washing steps to remove nonspecifically adhered biotin strands and incubations to allow binding of the free-end biotins with horseradish peroxidase (HRP) conjugated streptavidin. The oxidization of the added TMB substrates occurred, leading to the generation of visible color changes. Measuring the signal intensities at 450 nm showed that only in the presence of both the capture probe and the complementary oligo did the OD values exceed 3.0, while in the presence of the complementary probe alone, the OD readings fell below 0.3 (Figure 3(BII)). Such findings consolidate the derivation of the desired FLT3-targeting ssDNA probe appended with poly(A) tails for successful immobilization on gold-coated chip surfaces.

### 3.4. Structural and Morphological Analysis of Tailored Sensing Surface

**Figure 4:**
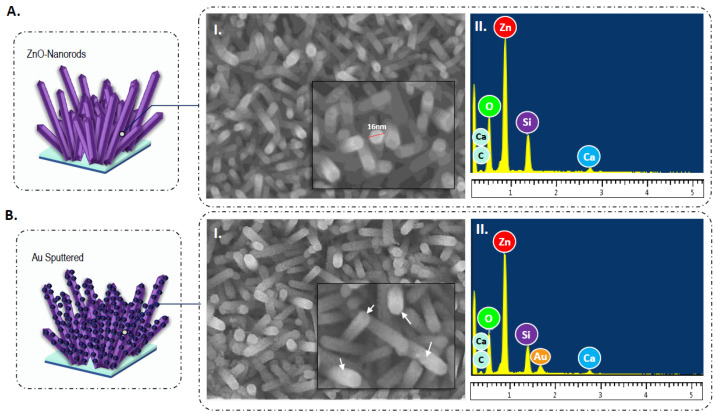
FESEM imaging of the surface grown (**A**(**I**)) ZnO-nanorods and (**B**(**I**)) ZnO-nanorods sputtered with gold with their analyzed EDX spectrum (**A**(**II**)) and (**B**(**II**)), respectively.

The surface morphology of the Au-sputtered ZnO-nanorods observed through FESEM imaging depicts a complete formation of ZnO nanostructures (Figure 4A,B). The nanorods are estimated to be in the width of 16–20 nm and possess hexagonal-shaped tips with a random pattern of nucleation. The absence of large particulate clumps together with the uniform coating of ZnO nanoparticles on the surface of the chips without any visible pores or pin-hole formations suggests the effectiveness of the ZnO spin coating and hydrothermal growth processes. Comparison of the morphological features of ZnO-nanorods with and without Au sputtering reveals the agglomeration of gold particles on top of the surface of the nanorods, resulting in a coarsened appearance of the film matrix. The adherence of Au together with ZnO nanorods substantially increases the surface area, which is expected to improve both the chemisorptive properties of the film and the accommodation of DNA molecules for successive probe immobilization and target hybridization stages [27]. An EDX spectrum of the constructed transducer film presents the elemental composition of the materials (Figure 4(AII,BII)). The spectrum exhibited peaks which showed the presence of oxygen (O), zinc (Zn), and gold (Au), further validating the deposition of ZnO and Au nanoparticles from the fabrication steps. The results also indicated the film/nanostructures being free of contaminants or impurities.

**Figure 5:**
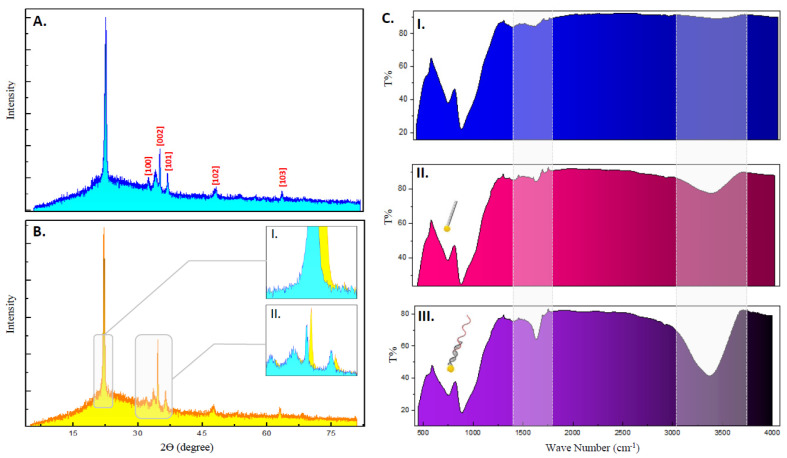
XRD diffractogram of (**A**) pure ZnO-nanorods and (**B**) Au-sputtered ZnO-nanorods. (**B**) (inset **I**,**II**) shows shifts in the XRD spectrum between pure and Au-sputtered nanorods. (**C**) The FTIR spectrum for Au-sputtered ZnO-nanorods (**I**), thiolated poly(T) strand-immobilized surface (**II**), and surface immobilized with poly(T) oligo duplexed FLT3 capture probe (**III**).

The quality of the surface-coated matrix such as their crystallinity, grain size, and preferential growth orientation was determined through XRD-based structural characterization. The diffraction peaks observed in the XRD diffractogram for the pure and Au sputtered ZnO-nanorods (Figure 5A,B) are in good accordance with the reference peaks shown in JCPDS cards for ZnO (ZnO JCPDS Card No. 36-1451). XRD analysis of ZnO exhibited narrow peaks at 31.02° (100), 34.40° (002), 36.31° (101), 47.65° (102), and 62.89° (103), indicative of their wurtzite phase, high crystallinity, and c-axis orientations (c-constant: 1.018, a-constant: 0.568; c > a). A more distinctive, dominant peak was further identified for pure ZnO-nanorods at angle 21.7°, insisting on the existence of nanosized and uniform crystallite growth orientations. However, the reflectance peaks for Au were absent within the spectra of Au-sputtered nanorods. This could be possible due to the small time window of sputtering applied during gold deposition, which results in a thin layer that is undetected during analysis as the XRD peaks for Au are more apparent at sputtering periods greater than 2 min [34,35,36]. The crystallite size of ZnO was computed to be 17.4 nm using Scherrer equations. 

It is also noticeable that sputtering Au onto the ZnO matrix induced numerous structural changes reflected within the XRD spectrum. The intensity of the peak (002) of ZnO increased for the gold-doped matrix with a broader curve area owing to dopant-induced disorders in the d-interspacing between the ZnO crystal lattice. Furthermore, the diffraction peaks for Au-ZnO complex shifted towards higher Bragg angles (Figure 5(BI,II)) insinuating the incorporation of Au dopants into the nanostructure [37]. Such inferences were evidently seen when the calculated crystallite size of Au-sputtered ZnO changed to 19.8 nm and after a decrease in the c-axis lattice constant (c-constant: 0.835), leading to improved grain boundaries and therefore better electrical conductivity [38]. Additional properties such as the dislocation density, which corresponds to the structural quality of the grown ZnO-nanorods, were elucidated using Equation (1), where *D* is the crystallite size and δ is the dislocation density defect. The values calculated for both pure and Au-sputtered ZnO-nanorods yielded values at 10^−3^, (ZnO:0.003 and Au-ZnO:0.0024), signifying the compactness of the grown matrix with fewer structural defects contributing to better film integrity and lower electrical resistance [39].

(1) $$\delta =\frac{1}{D^{2}}$$

FTIR spectroscopy was performed on probe-functionalized Au-sputtered ZnO-nanorods to ascertain the efficiency of DNA immobilization on the sensor platforms. The spectrum from the coated surfaces in the presence of DNA strands registered multiple absorption peaks absent in the non-DNA immobilized surfaces (Figure 5(CI–III)). These peaks attribute to the different functional groups and bond stretching that occurs during DNA tethering. A strong absorption peak was visible at regions between 1400 and 1700 nm, indicating the presence of groups and bonds of immobilized nucleic acid moieties, the fingerprint region for DNA molecules [40]. Absorption peaks at 1418 and 1640 cm^−1^ in the spectra correspond to the functional bases found in DNA strands such as cytosine, thymine, and guanine [41]. The absorption peak within the spectral regions between 3200 and 3800 cm^−1^ indicates the presence of N-H stretching vibration of purine and pyrimidines rings in DNA bases [42,43]. A decreasing trend in the intensity of transmittance of the FTIR spectrum was additionally observed for the immobilized ssDNA probes in comparison with the immobilized poly(T) tail surface. Such observations reveal the hybridization between the probe and the thiolated T-tail sequence, therefore contributing to a higher number of DNA molecules which translates to lower degrees of transmittance. This concurrently confirms the thiol-duplexing of the generated ssDNA probes as well as a successful thiol-gold mediated probe immobilization. It should be noted that the current study is different in several aspects compared with our previously reported sensor results [18], such as in the method of gold-incorporations and the size of the ZnO crystallites synthesized. The current study employed the use of a sputtering manner of Au deposition on to the sensing surface while the previous one utilized colloidal gold nanoparticles mixed into the ZnO sol-gel prior to coating. The grain size of the ZnO nanorods was also smaller than those fabricated in the previous study (30–40 nm), possibly due to the inverted manner of nanorod growth during hydrothermal incubations that may impart crucially different electrical and conductive properties to the proposed sensor.

### 3.5. Analysis of FLT3 Mutations through Electrochemical Impedance Spectroscopy

**Figure 6:**
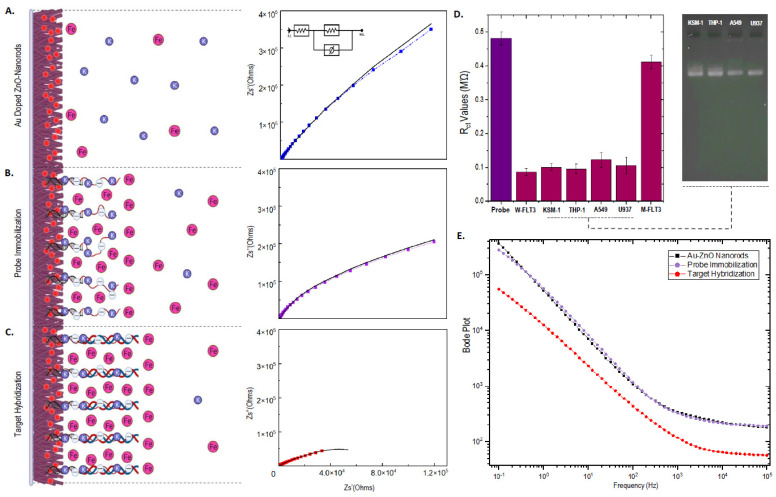
Schematic illustrations showing electrochemical behaviour on top the sensor surface and the surrounding electrolyte solutions with corresponding EIS spectrums obtained during measurement of (**A**) Au-sputtered ZnO-nanorods, (**B**) probe immobilization, and (**C**) target hybridization. (**D**) Bar chart of R_ct_ values measured for FLT3 target sequence obtained from different cell lines and with mutated FLT3 gene sequence, included with gel image of normal FLT3 from AML and non-AML cell lines. (**E**) Bode magnitude plot for the different stages of chip fabrication and target hybridization.

The electrochemical and surface charge properties of the designed sensor system were characterized using electrochemical impedance spectroscopy (EIS) with 2 mM of K_3_(Fe(CN)_6_)/K_4_(Fe(CN)_6_) electrolyte solution. The obtained Nyquist plot from each of the EIS measurements was fitted with the simple Randles equivalent circuit, enabling subsequent derivation of electrical parameters such as the surface charge transfer resistance (R_ct_). Constant phase element (CPE) was used instead of pure capacitance as this can account for surface heterogeneity caused by sputtering of Au atoms onto the ZnO matrix to provide a better curve fit, thus circumventing reduced surface uniformity and variation in signal relaxation times [27,44,45]. The R_ct_ values computed from the curve fitting of the Nyquist plots represent the resistance against the electron-transfer potential of the redox probe (ferricyanide) in the surrounding droplet of electrolyte solution towards the modified sensing region of the IDE. The changes in the surface-dependent R_ct_ values for the nanorod coated surfaces, including probe immobilization and target hybridization stages are tabulated (Table 2) and well portrayed in the obtained impedance spectrum, presented in Figure 6A–C. It is important to note that pre-sample processing steps such as extraction and precipitation of DNA are required prior to sample analysis. Pre-processing of AML cell samples also aids in removing any interferences contributed by protein-based analytes. Furthermore, the surface of the IDE biosensor being modified with vertical-rod-like ZnO nanostructures provides an uneven surface area that can easily disrupt any residual protein adsorptions, leading to accurate EIS signal readouts.

As inferred from the diagram, the EIS spectrum corresponding to the IDE sensor coated with Au-sputtered ZnO-nanorods yielded R_ct_ values around ~396 MΩ (Figure 6A). Such high degrees of resistance can be explained in terms of n and p-type semiconductor interactions at a quantum level. Adsorption of oxygen atoms at the ZnO surface during processes such as annealing creates a current/flow of electrons from the ZnO matrix towards the O_2_ atoms, causing the ZnO layers to harbor greater resistance. These O_2_-entrapped electrons induce spatial charge formations which create local electric fields that further impede film conductivity [35]. The larger surface area of the grown ZnO nanorods paired with their significantly higher grain size boundary causes even greater resistance since it leads to an increased amount of surface adsorbed O_2_ atoms and subsequent greater restriction in the movement of spatially trapped conduction band electrons. During sputtering of Au into the ZnO, Au atoms exist at higher Fermi energy levels due to their lower work functions [27,46], resulting in the flow of electrons from Au to the ZnO layer. This electronic transition to the ZnO layers possessing lower conductivity creates pn-junctions at the Au/ZnO interface, in turn improving the conductivity of the film. However, the degree of ohmic contacts between the Au/ZnO junctions could be greatly lower in this case due to the significantly smaller grain size of ZnO and the thin layer of sputtered gold atoms. This will attribute to both larger grain boundaries and smaller contact points for electron transitions, resulting in higher electrical resistance of the matrix. Such combinatorial factors can potentially contribute to higher surface impedance characteristics during EIS measurements [47].

During probe immobilization, the interaction of the FLT3-targeting ssDNA probe with the gold-sputtered ZnO-nanorod surface, mediated through thiol-gold bond formation, gave rise to an exponential decrease in R_ct_ values, recorded around ~0.44 MΩ (Figure 6B). We speculate that such a phenomenon can be caused by excessive counterion condensation at high surface probe concentrations used during probe immobilization (~10 µM–10 ug of ssDNA in 20 µL of buffer solution) [48,49]. The negatively charged phosphate backbone of immobilized FLT3 DNA probes creates a layer of accumulated negative charges in close proximity with the gold-sputtered ZnO surface, inevitably inducing strong ionic interactions with the counterions (K^+^) in the electrolyte solutions. The resultant cationic condensation could potentially redistribute the charge of the DNA probe layer to slightly positive values, causing a strong influx of the redox probes towards the gold-sputtered surface at a faster rate, corresponding to a drastic drop in R_ct_ values. This is because, if the DNA probe layer still possesses an overall negative potential or has a neutral charge due to charge neutralization by cationic (K^+^) interactions, the rate of transfer of the redox probe will remain impeded, reflecting almost similar R_ct_ values obtained for the gold-sputtered ZnO nanorods without DNA strands. The counterion condensation is possibly even greater for the proposed system since the immobilized FLT3 probe is longer (80 bp) and hybridized with a 20 bp poly(T) tail, contributing greater degrees of negative charges. In addition, due to duplex-tagging, the DNA probe will also be partially in an upright manner while the remaining single-stranded portion simultaneously possesses a lower gyration radius in a solvent environment [50], preventing the ssDNA probes from randomly coiling and adhering/blocking the surface. Thus, due to both an increase in counterion condensation and a partial upright, alleviated assemblage of the capture probes, even larger hexacyanoferrate anions will be able to exhibit faster transfer rates towards the gold surface and register lower R_ct_ values. Moreover, it can be considered that the functionalization of the Au/ZnO layer with lengthy probe sequences at high surface concentrations results in the DNA strands acting as an integral layer with the Au-ZnO nanorods instead of as separate biolayers. The observed decreases in the R_ct_ values could reflect off the ‘whole’ Au/ZnO-ssDNA nanorod film, in which the charged layer induces a faster rate of electron flow towards the Au/ZnO surface, given the occurrence of counterion condensations. This could be a highly plausible reason for the reduced R_ct_ values following probe binding even for Au/ZnO nanorods with high impedance traits.

The surface transfer resistance experiences further reductions at the stage of target hybridization at 1 µM of the FLT3 target sequence. Such readings are expected, as the binding of complementary DNA sequence cumulatively increases the net negative charge on the electrode surface. Moreover, the double-helix conformation assumed by the DNA strands during hybridizations further aids in decreasing steric hindrances for the ionic diffusion pathways. These factors amplify the anionic exchange, creating a greater influx of ions from the negative electrolyte layer and leading to lower R_ct_ values (~0.082 MΩ) and the downwards trends in the impedance spectrum with increasing target concentrations (Figure 6C). Similar results were obtained by Guler et al., verifying the results and explanations to be sound [51]. The bode magnitude plot (Figure 6E) corroborated the lower capacitance of both non-immobilized and ssDNA immobilized surfaces while displaying enhanced capacitive behaviors for target hybridized sensing surfaces due to both the increase in surface-layer thickness and surface-charge properties [52]. Intriguingly, the observed changes in the R_ct_ values did not correspond with the capacitance values recorded, specifically before and after probe immobilization. This could be due to the uneven surface of the nanorods preventing even distribution or alignment of the solvated ions and counterions compared with more flat electrode surfaces. The flexible probe layer on top the nanorods can also relatively assume repeating partial coils and extensions at the interfacial layer, further preventing even distributions of ions [51,53]. However, during target hybridization, the complete, upright structural assembly of dsDNA can help establish a more stable and rigid film layer, causing an even collection of negatively charged ions inferred from the drop in CPE values.

The ability of the biosensor to detect and differentiate unmutated and mutated forms of the FLT3 gene was ratified using targeted FLT3 sequence obtained from various sources. Here, the sensor platform was analyzed by allowing hybridization to occur with the target sequence of the wild-type FLT3 (W-FLT3) found in normal human cells. In addition, the system was also analyzed using AML cancer cells (Kasumi-1 and THP-1) and non-AML cancer cells (A549 and U937), both reported to harbor similar wild-type FLT3 sequences [54]. This is seen where PCR amplification of the normal FLT3 gene region from gDNA of all four cells line display bands of equal size (Figure 6D, gel image), supporting the presence of the same targeted gene in each sample. The R_ct_ values obtained for each of the different samples were observed as below ~0.12 MΩ (Figure 6D). This demonstrates the high selectivity and specificity of the designed capture probe to hybridize well with any unmutated FLT3 sequence processed from normal human cells, AML cancer cells independent of FLT3-ITD mutations, as well as with irrelevant cancer cells. To evaluate the change in the computed R_ct_ values for a mutant variation of the FLT3 gene, a probe sequence was utilized to mimic DNA hybridizations with samples possessing FLT3-ITD mutations. The mutant sequence constituted an 18 bp duplicated section of the gene which is known to undergo high frequencies of internal tandem duplications (5′—TTTTTTTAATTGCT[GGTACCATTCCGTGGTAT][GGTACCATTCCGTGGTAT]GAATAGATCTTATAATTTATTTTATCCATTATGTTCACGAAGCACAT—3′) [30]. The R_ct_ value obtained for the mutant gene sequence showed a significantly higher reading (~0.41 MΩ) closer to the values obtained during probe immobilization (Figure 6D). This implies the absence of a strong complementary binding with the target capture probe that can easily destabilize under stringent washing steps. Such resultant EIS signal responses measured from normal and mutant gene targets dictate the reverse sensing mechanism of the designed biosensor, facilitating accurate detections of AML cancers caused prominently by FLT3-ITD mutations. Under possible circumstances of false-positive signals, PCR or asymmetric-PCR (A-PCR) amplification steps of the FLT3 gene from AML samples can be performed/included in order to rectify such complications. By specifically amplifying the FLT3 gene as the initial DNA sample for the proposed sensor system, the degree of false-positive signals can be reduced to negligible levels. This helps eliminate off-target sequences which may also not bind to the capture probe and lead to false positives for such reverse detection sensors. The probe is also strategically and rationally designed to hybridize with both the normal portion and the region of the FLT3 gene which undergoes tandem duplications. Together with optimized binding and washing steps, the capability of the sensor system to specifically bind and differentiate normal and mutated FLT3 gene sequences will be precise and reliable. A key advantage in coupling DNA amplifications steps with sensor-based mutation detections is the possibility of developing multiplex sensor devices. Since mutations leading to the prognosis of AML progression is not limited to signal-pathway genes such as FLT3 only (e.g., KRAS, NRAS, KIT, PTPN11, and NF1), the proposed sensor system can be designed as a multiplexed panel of DNA probes targeting various DNA sequence mutations in a single platform, leading to concomitant identification of multiple mutations that can yield an entire gene mutation profile for AML patients [55,56].

### 3.6. Assessment of the Analytical Performance of AML Sensor

**Figure 7:**
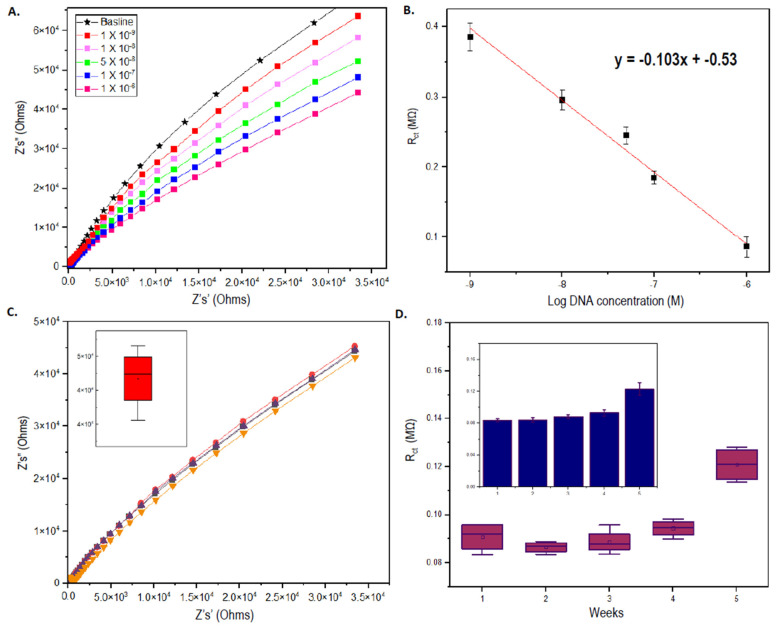
(**A**) Depicts the EIS signals obtained at each different target concentrations of FLT3 gene sequence. (**B**) Shows the plot of R_ct_ values against Log DNA concentrations. (**B**) (inset) shows the R_ct_ values at different surface probe concentrations (**C**) Shows the reproducibility of the sensor platform taken from four different sensor replicates. (**D**) Shows the stability of modified IDE sensors tested over 5 weeks.

The fabricated sensor’s analytical parameters were tested and interpreted accordingly (Figure 7A,B). The sensor exhibited good levels of sensitivity with a limit of detection at 1 nM (Figure 7A), estimated using signal-noise ratios more than 3 σ [57,58]. The R_ct_ value obtained for the lowest detectable sample concentration (~0.38 MΩ) was clearly distinguishable and statistically significant (*p* > 0.001, α = 0.05) from the baseline value, which is the R_ct_ recorded during probe immobilization. For further affirmation, obtained R_ct_ values of different target concentrations were computed with *t*-test analysis. The *p*-values obtained between each consecutive measurement during sensitivity analysis were less than the alpha values, which statistically proves the significant difference between each EIS measurement for individual analyte concentrations. Moreover, the value obtained for the lowest target concentration of the normal FLT3 gene sequence was also significantly lower than the R_ct_ response value obtained for the mutant target sequence (*p* > 0.03, α = 0.05), indicating the accuracy of the system to discriminate between normal and mutant FLT3 genes at nanomolar levels. Based on the plot of R_ct_ values against Log of target FLT3 DNA concentrations (Figure 7B), the linear fit of the values yielded an r^2^ equivalent to ~0.98, which shows the commendable linearity of the designed biosensor. The concentration of surface-immobilized ssDNA probes was rationally selected to be at 10 µM as the ideal concentration required to generate optimum signal outputs. Surface probe concentrations higher than 10 µM are not recommended, as they could potentially lead to surface probe entanglements due to a more densely packed ssDNA probe layer, skewing the accuracy of EIS signal measurements [50]. Concentrations below 10 µM such as 1–5 µM are more suitable for ssDNA probes with less than 25 bp, as they will permit greater numbers of DNA strands/µL of the buffer solution for better target binding. For longer DNA probe sequences (e.g., FLT3 probe of 80 bp), lower concentrations will reduce the number of DNA strands/µL of solutions, limiting their potential for maximum gene target interactions.

Reproducibility and stability tests conducted using 1 µM of FLT3 gene target also displayed favorable results. The sensor’s reproducibility was observed to be acceptable, having less than 5% of deviation among the four obtained readings that were prepared and measured under the same experimental conditions (Figure 7C). The sensor also possessed excellent long-term stability since the R_ct_ values underwent increment by only ~20% even during the 5th week of measurement, kept under 4 °C storage conditions (Figure 7D). It should be noted that the proposed sensor system displayed a high degree of selectivity against the complementary FLT3 gene while still being able to differentiate between mutant FLT3 targets having partial complementarity. However, testing the system with other non-specific DNA sequences is impractical as the signals measured from mutant targets and off-targets will overlap, leading to an unclear difference between true negative and false negative EIS signals. The response time for the sensor system was estimated to be around ~1 min, in reference to previously established similar sensing matrixes [27,59]. Nonetheless, since the sensor relies on non-transient, stable EIS signal readouts, the implications in determining the response time are less significant for such sensor applications. On another note, such systems which deploy the use of duplexed-tagging of capture probes to enable thiol-gold surface immobilization creates an amenable and flexible biointerface system. It is reported that during electrochemical measurements, discharges of lengthy, hybridized DNA strands from the surface have been recognized, especially for sensors which involve passive adsorptions [60,61]. The proposed system does not encounter such drawbacks as seen from the persisting stability of the sensing surfaces. However, immobilization strategies utilizing duplexed-tagging for the capture probe are immune towards such complications, as only the shorter poly(T) strands being inherently less susceptible to changes in the surface microenvironment are anchored to the Au-sputtered surface through thiol interactions. The longer portions of the FLT3 probes can be re-immobilized by reannealing them with the poly(T) oligos to account for induced release, thus “recharging” the biosensor and augmenting their reusability. Such a biointerfacing mechanism can also promote the use of different probes targeting different DNA gene targets, enabling the interchangeability of modified transducer film layers to facilitate different capture probes for the detection of different analytes as well.

## 4. Conclusions

Conclusively, we described here the development of an efficient DNA biosensor for the detection of FLT3-ITD mutations in AML-type DNA samples. The sensor implements a simple reverse sensing mechanism which enables accurate recognition of AML samples specifically bearing the FLT3-ITD mutations. The IDE-based sensor offers robust and sensitive analyte detections with reliable outputs in terms of reproducibility, reusability, and stability. In the future, such manner of systems can be adapted into multifaceted devices which can detect several different gene mutations associated with AML cases, generating a complete genetic profile of AML patients from a single nanosensor platform. The created nanobiosensor opens new promising routes in both clinical and diagnostic applications.

## Figures and Tables

**Table 1:** The sequence of primers with adapters, template, and DNA probe.

Label	Sequence (5′-3′)
Forward primer	TTTTTTTTAATTGCTGGTACCATTCCGTGGTATGAAT
Reverse primer	TTTTTTTTTTTTTTTTTTTTATGTGCTTCGTGAACATAATGGATAAAATA
Template	CCATTCCGTGGTATGAATAGATCTTATAATTTATTTTATCCATTATGTT
DNA Probe	TTTTTTTTAATTGCTGGTACCATTCCGTGGTATGAATAGATCTTATAATTTATTTTATCCAT TATGTTCACGAAGCACATAAAAAAAAAAAAAAAAAAAA

**Table 2:** Curve fit values for the Randles circuit of the impedance measurements.

Label	R_ct_ (Ω)	R_s_ (Ω)	*n*	Q (CPE)	Chi-Squared Value
Au/ZnO-Nanorods	3.27 × 10^8^	209.6	0.84	4.305 × 10^−6^	13.87 × 10^−3^
Au/ZnO-Nanorods/DNA probe (10 µM)	4.829 × 10^5^	208.4	0.84	3.746 × 10^−6^	4.871 × 10^−3^
Au/ZnO-Nanorod/DNA probe (10 µM)/target (1 µM)	8.65 × 10^4^	48.44	0.83	3.73 × 10^−5^	2.582 × 10^−3^
